# Next-generation humanized NSG-SGM3 mice are highly susceptible to *Staphylococcus aureus* infection

**DOI:** 10.3389/fimmu.2023.1127709

**Published:** 2023-03-10

**Authors:** Sophia Hung, Amelie Kasperkowitz, Florian Kurz, Liane Dreher, Joachim Diessner, Eslam S. Ibrahim, Stefan Schwarz, Knut Ohlsen, Tobias Hertlein

**Affiliations:** ^1^ Institute of Molecular Infection Biology, University of Würzburg, Würzburg, Germany; ^2^ Institute of Microbiology and Epizootics, Centre for Infection Medicine, School of Veterinary Medicine, Freie Universität Berlin, Berlin, Germany; ^3^ Veterinary Centre for Resistance Research (TZR), School of Veterinary Medicine, Freie Universität Berlin, Berlin, Germany; ^4^ Institute of Pathology, University of Würzburg, Würzburg, Germany; ^5^ Department for Obstetrics and Gynecology, University Hospital of Würzburg, Würzburg, Germany; ^6^ Department of Microbiology and Immunology, Faculty of Pharmacy, Cairo University, Cairo, Egypt

**Keywords:** humanized mice, *Staphylococcus aureus*, MRSA, NSG, NSG-SGM3, staphylococcal abscess, *Staphylococcus aureus* immune response, humanized hemato-lymphoid mice

## Abstract

Humanized hemato-lymphoid system mice, or humanized mice, emerged in recent years as a promising model to study the course of infection of human-adapted or human-specific pathogens. Though *Staphylococcus aureus* infects and colonizes a variety of species, it has nonetheless become one of the most successful human pathogens of our time with a wide armory of human-adapted virulence factors. Humanized mice showed increased vulnerability to *S. aureus* compared to wild type mice in a variety of clinically relevant disease models. Most of these studies employed humanized NSG (NOD-*scid* IL2Rg^null^) mice which are widely used in the scientific community, but show poor human myeloid cell reconstitution. Since this immune cell compartment plays a decisive role in the defense of the human immune system against *S. aureus*, we asked whether next-generation humanized mice, like NSG-SGM3 (NOD-scid IL2Rg^null^-3/GM/SF) with improved myeloid reconstitution, would prove to be more resistant to infection. To our surprise, we found the contrary when we infected humanized NSG-SGM3 (huSGM3) mice with *S. aureus*: although they had stronger human immune cell engraftment than humanized NSG mice, particularly in the myeloid compartment, they displayed even more pronounced vulnerability to *S. aureus* infection. HuSGM3 mice had overall higher numbers of human T cells, B cells, neutrophils and monocytes in the blood and the spleen. This was accompanied by elevated levels of pro-inflammatory human cytokines in the blood of huSGM3 mice. We further identified that the impaired survival of huSGM3 mice was not linked to higher bacterial burden nor to differences in the murine immune cell repertoire. Conversely, we could demonstrate a correlation of the rate of humanization and the severity of infection. Collectively, this study suggests a detrimental effect of the human immune system in humanized mice upon encounter with *S. aureus* which might help to guide future therapy approaches and analysis of virulence mechanisms.

## Introduction

The evaluation of promising new treatments against infectious diseases is challenging and the path to clinical application is plastered with failures. *Staphylococcus aureus* has emerged as a prominent example in this group. This bacterial pathogen causes a wide array of diseases, ranging from superficial skin infections to life threatening bacteremia, endocarditis, pneumonia and osteomyelitis (1–3). Its genetic flexibility, evolution of host-specific virulence factors and notorious acquisition of antimicrobial resistance genes makes it one of the most important bacterial pathogens of our time (4, 5).

Despite huge clinical and economic impact, all immunotherapies - most importantly vaccination attempts - have so far failed (6, 7). Multiple reasons for the lacking efficacy of these approaches during clinical trials, although being effective in pre-clinical models, have been proposed by the scientific community with two standing out: (I) the lack of understanding of host-pathogen interaction during infection in humans and (II) the poor translational power of pre-clinical data (7, 8).

Humanized mice, or humanized hemato-lymphoid system mice, have drawn attention in recent years as a promising solution to at least some of these problems (9). This model is based on highly immunodeficient mouse strains which are engrafted with human hematopoietic stem cells, which in turn differentiate into various human immune cell lineages in these mice (10, 11). This makes it possible to investigate host-pathogen interaction as well as the interplay of different human immune cell populations in a highly complex *in vivo* system.

Recent publications in this field suggest that humanized mice are an interesting and viable option to investigate *S. aureus* infection. They show that humanized mice are much more susceptible to *S. aureus* infection than wild-type, murinized (immunodeficient mice with engrafted murine stem cells) and even non-engrafted immunodeficient mice in models of peritonitis (12), pneumonia (13), skin infection (14), osteomyelitis (15) and deep tissue infection (16). These studies furthermore delivered proof, that some virulence factors of *S. aureus* show much higher activity against human than murine cells and factors during *in vivo* infection (13, 14). Nonetheless, it has to be stated that the humanized mouse models studied thus far harbor a serious drawback, particularly in studying *S. aureus* infection: the numbers of human myeloid and monocytic cells are rather low. This is due to the application of NSG mice, which are widely used in the field, but are unable to sustain high numbers of these immune cells after engraftment due to the lack of necessary human growth factors and cytokines (17, 18). Several strategies have been applied to overcome this problem: the administration of these factors during humanization (19, 20), hydrodynamic injection of human cytokine-encoding plasmids (21) or genetically engineered humanized mice, which are producing these factors (22, 23).

Since neutrophils and the macrophage-monocyte axis play a prominent role during the immune response against *S. aureus* (24–26), we asked whether next-generation humanized mice perform better or worse during *S. aureus* infection than the above-mentioned humanized NSG mice. Therefore, we humanized NSG-SGM3, which provide high numbers of human monocytes and neutrophils due to genetical integration of human SCF, GM-CSF and IL-3 genes (17), and compared their performance during *S. aureus* deep tissue infection with those of humanized NSG mice.

## Materials and methods

### Ethics statement

All animal studies were approved by the local government of Lower Franconia, Germany (approval numbers 55.2-2532-2-836 and 55.2-2532-2-1129) and performed in strict accordance with the guidelines for animal care and experimentation of the German Animal Protection Law and the DIRECTIVE 2010/63/EU of the EU. The animals were housed in IVC cages under standardized lighting conditions and had *ad libitum* access to food and water. All experimentations with anonymized and non-traceable human cord blood was approved by the ethics committee of the University Wuerzburg (approval number 20191212 02).

### Humanization and murinization procedure (incl. CD34+ cell isolation)

We included female and male NSG (NOD.Cg-*Prkdc^scid^ Il2rg^tm1Wjl^*/SzJ) and NSG-SGM3 (NOD.Cg-*Prkdc^scid^ Il2rg^tm1Wjl^* Tg(CMV-IL3,CSF2,KITLG)1Eav/MloySzJ) mice from the Jackson Laboratories (Bar Harbor, ME, USA) and female Balb/c mice (BALB/cJRj, Janvier labs, Le Genest-Saint-Isle, France) in all experiments. The human CD34+ hematopoietic stem cells were isolated from human cord blood by magnetic separation (EasySep™ Human Cord Blood CD34 Positive Selection Kit II, STEMCELL technologies, Cologne, Germany) following the manufacturer’s protocol. The quality of the cell preparation was controlled by staining for hCD34+ and hCD3+ cell markers by flow cytometry. Only preparations with > 85% hCD34+ purity and < 1% hCD3+ cell content were used for humanization purposes. Humanized NSG (huNSG) and NSG-SMG3 (huSGM3) mice were generated by engrafting 100,000 hCD34+ cells (of a donor mix) at the age of 6 – 8 weeks, similar to the procedures described in earlier publications (15–17, 27). Briefly, after whole-body irradiation with a sub-lethal dose of 2 Gy, mice were injected intravenously with the human hematopoietic stem cells. Murinized NSG-SGM3 mice (muSGM3) received a similar treatment than huNSG and huSGM3 mice, but 100,000 bone marrow cells from a Balb/c donor instead of human CD34+ cells were injected. The peripheral blood of humanized mice was analyzed every two weeks after engraftment for the presence and frequency of murine CD45+, as well as human CD45+, CD66b+, CD3+ and CD20+ cells by flow cytometry.

### Determination of blood hemoglobin content and erythrocyte and reticulocyte numbers

Every two weeks during the course of the humanization and at the end of the bacterial thigh muscle infection, peripheral blood was collected and mixed with EDTA as anticoagulant (pluriSelect Life Science, Leipzig, Germany). The hemoglobin content (Hämoglobin, Diaglobal GmbH, Berlin, Germany) and the red blood cell count (Erythrozyten Gower’s Reagenz, Bioanalytic GmbH, Umkirch/Freiburg, Germany) was measured by fluorometric assays following manufacturer’s instructions. During the humanization period, we furthermore determined the reticulocyte numbers by streaking blood samples on glass slides and counted the number of reticulocytes among red blood cells after staining (Brillant cresyl blue, Bioanalytic GmbH, Umkirch/Freiburg, Germany).

### Thigh infection model (including determination of bacterial burden)

HuSGM3 mice were infected intramuscularly (i.m.) at 12 weeks and the huNSG mice at 18 weeks post hCD34+ stem cell injection as described previously (16). At these points human CD45+ cell numbers reached robust numbers in blood and the number of hCD3+ T cells and hCD19+ B cells were at comparable levels. Balb/c, wild-type NSG-SGM3 and murinized NSG-SGM3 mice were infected at the age of 18 weeks in order to match the age of the humanized NSG-SGM3 mice (engraftment at the age of approximately 6 weeks and 12 weeks of humanization). Briefly, Methicillin-resistant *S. aureus* (MRSA) LAC* *lux* (28) was pelleted after overnight shaking at 37°C in B medium and resuspended in 0.9% NaCl solution. The left thigh of each mouse was then shaved, disinfected and injected with 1 x 10^8^ CFU bacteria in a total volume of 50 µL (16, 29). Besides huSGM3 and huNSG mice, we used age-matched murinized NSG-SGM3, wild-type NSG-SGM3 and Balb/c mice as controls. The wellbeing of each mouse was inspected and scored every 12 hours p.i. and the weight measured every 24 hours. Those mice that did not reach the humane end point as defined by the score sheet were either sacrificed on day 2 or on day 7 p.i. Peripheral blood, the infected thigh muscle, kidneys, liver, spleen, heart, lung and bone marrow from tibia and femur were then harvested. The spleens were halved and one part homogenized by pressing through a cell strainer for flow cytometry and bacterial burden determination, while the second half was processed for histological examination. The thigh muscle, kidneys, liver, lung and heart were homogenized in 0.9% NaCl and serial dilutions were plated on B agar plates in order to determine the bacterial burden. Bone marrow was harvested by flushing both femurs and tibias with sterile 0.9% NaCl solution, followed by filtration through a cell strainer.

### Histology

Histological sections and immunohistochemical stainings of splenic tissue were performed using formalin-fixed and paraffin-embedded (FFPE) tissue slides according to standard protocols. Briefly, spleens were fixed overnight in 10% neutral-buffered formalin solution, embedded in paraffin and cut to 5 µm slices. After deparaffinization, samples were stained with H&E and immunohistochemistry staining was performed with anti-human CD45 primary antibody (Agilent Dako, Waldbronn, Germany) at the Institute of Pathology at the University Clinics Wuerzburg according to the appropriate protocols within an automated immunostainer (Benchmark Ultra; Ventana/Roche, Tucson AZ, USA). Specimens were then inspected by an experienced pathologist (FK) for the presence and organization of human immune cells in the spleens.

### Flow cytometry

The peripheral blood of humanized mice was examined by flow cytometry every two weeks after stem cell administration. Blood samples were therefore stained with anti-human CD45/CD3/CD19/CD66b and anti-mouse CD45. The rate of humanization was calculated as: hCD45+ cells/(hCD45+ cells & mCD45+ cells), as applied previously (16). The peripheral blood, the spleen homogenate and bone marrow were interrogated for the presence of immune cells on day 2 p.i. with combinations of anti-human CD45/CD3/CD19/CD14/CD66b and anti-mouse CD45/Ly6C/Ly6G antibodies after red blood cell lysis. All antibodies were supplied by Miltenyi Biotec (Bergisch Gladbach, Germany). Flow cytometric measurements were performed on a MACSQuant flow cytometer and analyzed with MACSQuantify software 2.6 (Miltenyi Biotec, Bergisch Gladbach, Germany).

### Determination of cytokine levels and myeloperoxidase activity

In order to determine cytokine levels in the thigh muscle, homogenate was centrifuged at 3,000 x g for 5 minutes and the supernatant stored at -80°C until further processing. The peripheral blood samples were agglutinated overnight at 4°C, then centrifuged at 15,000 x g for 15 minutes. The serum was harvested and stored at -80°C until cytokine measurement. Levels of human or murine CCL-2, IL-1β, IL-6, IL-10, IL-17A, and TNF-α as well as human IL-8 were measured in the infected thigh muscles or in peripheral blood with a custom-mixed Luminex assays from Bio-Techne (Wiesbaden, Germany) following the manufacturer’s manual. In order to test the specificity of the individual assay, we measured the cytokine standards of the human kit with the mouse kit and vice versa and could not detect values above background levels.

### Statistical analyses

All statistical analyses were performed with GraphPad Prism (9.1.2) and p < 0.05 was considered as significant. The applied statistical tests can be found in the respective figure caption.

## Results

### Humanized NSG-SGM3 mice show strong human immune cell engraftment and develop anemia during prolonged course of humanization

**Figure 1:**
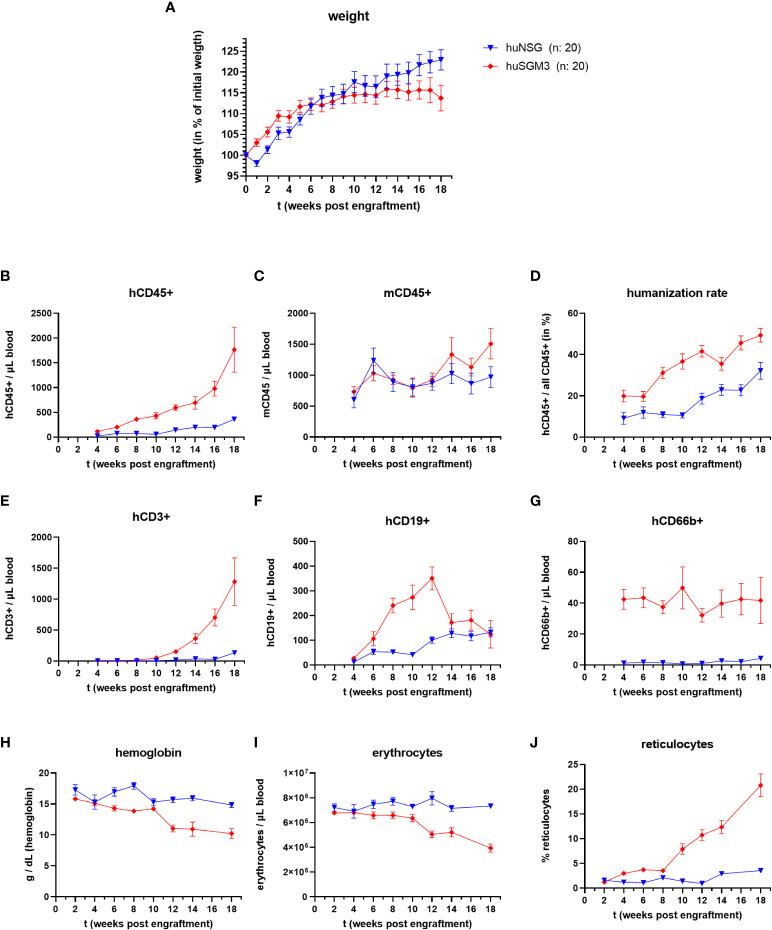
Weight, immune cell types and blood cells during the course of humanization of NSG and NSG-SGM3 mice. NSG or NSG-SGM3 mice were humanized by intravenous administration of 100.000 cord-blood derived hCD34+ cells. **(A)** The weight gain of each mouse compared to their weight on the day of engraftment was calculated and the mean values +/- SEM of each group is displayed. Statistical differences between both mouse strains was determined with Mann-Whitney test for each respective point in time. **(B–H)** The blood cell numbers of human and murine CD45+ cells, as well as human T cells **(E)**, B cells **(F)** and granulocytes **(G)** were determined every two weeks post engraftment by flow cytometry. **(D)** The humanization rate was calculated as hCD45+/(hCD45+ and mCD45+) cells. Visualized are the mean values +/- SEM for each group. **(H–J)** The level of hemoglobin, as well as the numbers of erythrocytes and the percentage of reticulocytes was measured bi-weekly and is displayed as mean +/- SEM for each group.

We generated humanized NSG (huNSG) and NSG-SGM3 (huSGM3) mice by sublethally irradiating the animals and intravenously injecting human cord blood-derived CD34+ hematopoietic stem cells. The mice were then continuously monitored and weighed over a period of 18 weeks. A divergence between the two groups in terms of body weight change (**Figure 1A**) became obvious. While huNSG mice continually gained weight, huSGM3 mice initially gained weight, too, but then started to stagnate from week 12 to 18.

Overall, huNSG and huSGM3 mice showed similar developments and levels of humanization rates and individual immune cell populations than described earlier (16, 30). HuSGM3 animals had throughout the whole course of humanization a higher rate of humanization and stronger increase in human immune cells (**Figures 1B–F**). In particular, the amount of hCD66b+ cells was at all times strongly enhanced compared to huNSG mice.

Since symptoms of secondary hemophagocytic lymphohistiocytosis (HLH) and/or macrophage activation syndrome (MAS) were described for NSG-SGM3 mice after engraftment with human CD34+ stem cells (30–32), we measured the numbers of erythrocytes, reticulocytes and the level of hemoglobin in the blood of the humanized mice every two weeks post engraftment (**Figures 1H–J**). While all three parameters remained at a comparable level for the huNSG mice, we saw a drop in erythrocyte numbers and hemoglobin levels as well as an increase in reticulocyte numbers in huSGM3 mice during prolonged course of humanization. The development and extend of these changes fits to earlier description and started at 8 to 14 weeks post engraftment (30–32). Of note, this was accompanied by a strong acceleration of hCD3+ T cell numbers in blood. The reticulocytosis implies that this is caused by a decimation or increased usage of erythrocytes, not a dysfunctional production (30, 31).

### MRSA thigh infection leads to strongly reduced survival of huSGM3 mice

**Figure 2:**
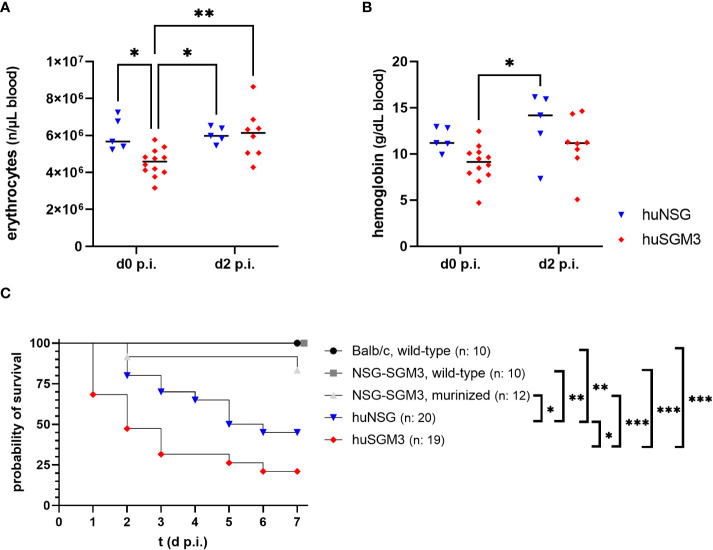
Erythrocyte numbers, hemoglobin levels and survival during severe local *S. aureus* infection in the thigh muscle of mice. The number of erythrocytes **(A)**, as well as the amount of hemoglobin **(B)** was measured fluorometrically at the start and on day 2 after infection with 1 x 10^8^ *S. aureus* LAC* lux. Individual values per mouse as well as the respective medians per group are displayed. Statistical significance was tested with an ordinary two-way-ANOVA and Sìdàk’s multiple comparison test (*p < 0.05, **p < 0.01). **(C)** The survival curve displays the percentage of animals per group which did not reach the humane end point as determined by the score sheet at the respective time. Statistically significant differences between the group survival was determined with Gehan-Breslow-Wilcoxon-test (*p < 0.05, **p < 0.01, ***p < 0.005).

Based on the results from the humanization phase, we decided to infect huNSG mice at 18 weeks and huSGM3 at 12 weeks after engraftment. HuSGM3 mice showed at this point an overall enhanced level of human immune cells compared to huNSG mice, with slightly reduced levels of hemoglobin and erythrocytes and increased numbers of reticulocytes. Both humanized mouse groups were infected locally with 1 x 10^8^ CFU *S. aureus* LAC* *lux* in the left thigh muscle, with wild type NSG-SGM3, murinized NSG-SGM3 (which were treated equally to humanized NSG-SGM3, but received murine bone marrow cells instead of human CD34+ stem cells) and wild type Balb/c mice as controls. This type of infection causes the formation of large deep tissue abscesses in wild-type mice as described earlier (29). Since *S. aureus* utilizes various mechanisms and virulence factors to acquire iron from hemoglobin (33, 34), we decided to track the levels of erythrocytes and hemoglobin in the humanized mouse groups during the course of infection. The overall amounts were, for both factors, lower in huSGM3 mice than in huNSG mice at the start of infection (**Figures 2A, B**). But the level of erythrocytes in the blood of huSGM3 mice increased in the first 48 hours of infection, reaching the levels of huNSG.

Mice were weighed and inspected every 12 hours throughout the infection experiment and received a score based on weight loss and signs of disease. The humanized mouse groups proved to be much more susceptible to the deep tissue infection with *S. aureus* than the control groups (**Figure 2C**), which fitted to earlier results in this model (16). Nonetheless surprising was the extreme vulnerability of next-generation humanized (huSGM3) mice which showed early in the experiment very strong signs of disease and weight loss, causing a strongly decreased survival rate compared to all other groups, even significantly inferior to huNSG.

### Bacterial burden in the thigh muscle and inner organs of infected mice

**Figure 3:**
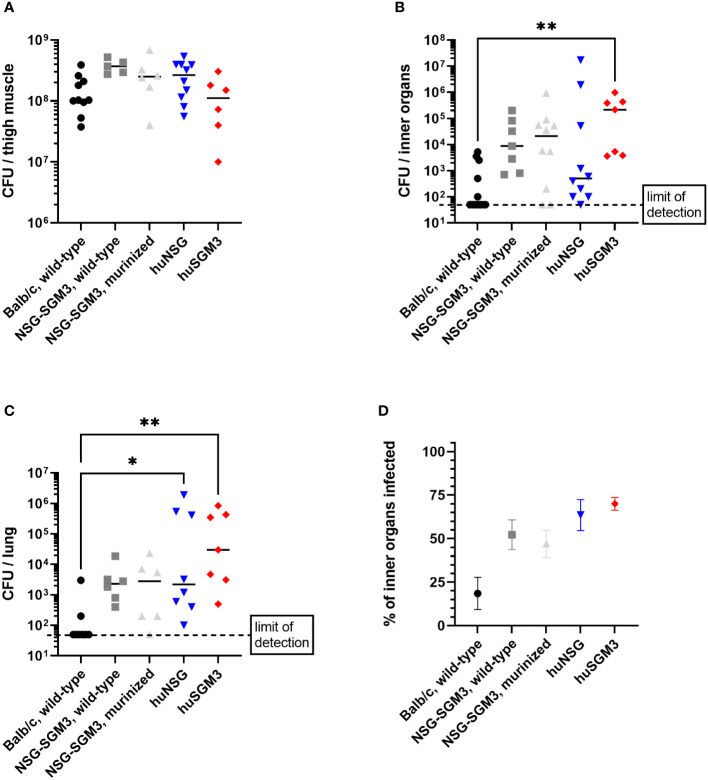
Bacterial burden in *S. aureus*-infected mice on day 2 p.i. The infected thigh muscle, as well as liver, kidneys, heart and lung were recovered on day 2 p.i. and homogenized. Serial dilutions were then plated to measure the colony forming units (CFU) of *S. aureus* in the respective organ. Displayed are the individual bacterial burden in the thigh muscle **(A)**, the lung **(C)** or the combined bacterial burden in investigated inner organs (kidneys, liver, heart, spleen and lung) **(B)**. The percentage of infected inner organs was furthermore calculated for each individual mouse **(D)**. Statistical significance was tested with Kruskal-Wallis with Dunn’s multiple comparison test (*p < 0.05, **p < 0.01).

The strongly reduced survival rate of huSGM3 mice raised the question of the cause for the enhanced vulnerability, particularly in comparison to huNSG mice. In a first attempt to define the systemic consequences of a local infection with *S. aureus* in the thigh muscle, we analyzed the bacterial burden in various organs on day 2 p.i. Later dates were not accessible since the number of surviving huSGM3 mice was not sufficient for analysis. No difference in terms of bacterial burden could be seen at the primary site of infection (**Figure 3A**). The analysis of bacterial burden in kidneys, liver, lung, spleen and heart revealed, that humanized mice showed a tendency towards higher numbers of *S. aureus* in the respective organs, although the differences were rather small and the results varied (**Figure 3B**). The strongest difference could be measured in the lungs with humanized mice displaying significantly higher bacterial numbers than Balb/c mice (**Figure 3C**). Balb/c mice showed the weakest bacterial spreading and infection of inner organs, while it was similar for all other groups (**Figure 3D**). Overall, a clear correlation of bacterial burden on day 2 p.i. and survival during the course of infection could not be established.

### Stronger response of human immune cells in humanized NSG-SGM3 than in humanized NSG mice

**Figure 4:**
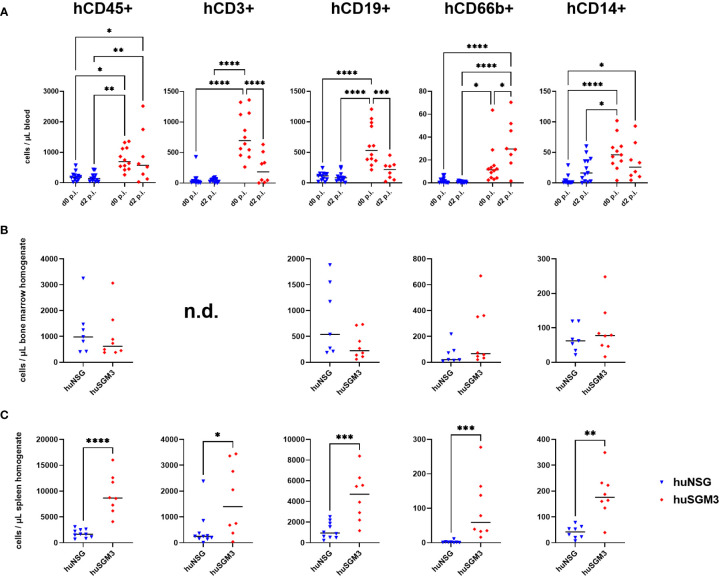
Human immune cell types in *S. aureus*-infected huNSG and huSGM3 mice. Immune cells were measured by flow cytometry with antibodies against hCD45, hCD3, hCD19, hCD66b and hCD14. **(A)** Blood samples were drawn at the start point of infection and on day 2 p.i. **(B)** Bone marrow was harvested by flushing tibia and femur on day 2 p.i. **(C)** Spleens were recovered on day 2 p.i. and homogenized by pressing through a 70 µm cell strainer. Displayed are the individual values per mouse as well as the medians per group. Statistical significance was tested with either Kruskal-Wallis with Dunn’s multiple comparison test **(A)** or Mann-Whitney-test **(B** + **C)** (*p < 0.05, **p < 0.01, ***p < 0.005, ****p < 0.001).

**Figure 5:**
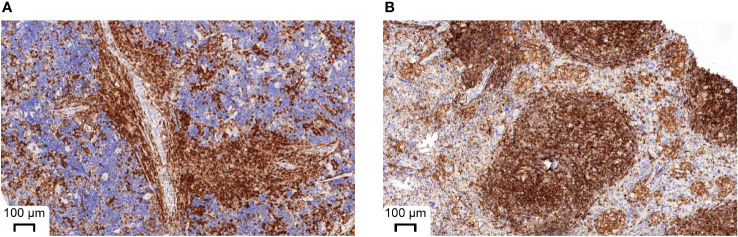
Representative histological appearance of spleens from huNSG-SGM3 **(A)** and huNSG mice **(B)** during *S. aureus* infection. Spleens were harvested on day 2 p.i. and processed to formalin-fixed and paraffin-embedded (FFPE) tissue slices. Specimens were then stained with anti-human CD45 and H&E. Strong CD45 expression can be seen at the periarteriolar lymphoid sheath and associated lymph follicles.

Since the number of bacteria during infection did not coincide with the severity of infection, we next analyzed immune cell types and effector molecules in order to identify differences which might explain the high vulnerability of huSGM3 mice. First, we compared major murine immune cell populations in the blood, spleen and bone marrow of all mice (**Figure S1**). Both humanized mouse groups had similar numbers of murine CD45+ cells, granulocytes and monocytes, but significantly less than wild type or murinized mouse groups (**Figure S2**). Immunophenotyping of human immune cells in the blood of huSGM3 and huNSG mice at the start of the infection and on d2 p.i. showed overall higher numbers of all investigated cell types in huSGM3 mice, namely T cells, B cells, granulocytes and monocytes (**Figure 4A**). The numbers remained stable in huNSG within the first 2 days of infection, but a clearly distinguishable pattern became visible for the huSGM3 mice. While the number of hCD66b+ granulocytes increased between d0 and d2 p.i., we could see significant drops of B and T cell numbers in the blood. The pattern of higher human immune cell numbers in huSGM3 mice was interestingly mirrored in the spleen (**Figure 4C**) but not the bone marrow (**Figure 4B**) on d2 p.i. The number of human cells in general, as well as of B cells, monocytes and granulocytes in particular, were similar in the bone marrow of huSGM3 and huNSG mice. In contrast to this, we could measure five-fold more hCD45+ cells in the spleens of huSGM3 mice than in huNSG mice, with all investigated human immune cell types represented significantly stronger. Histological examination of the spleens from huNSG and huSGM3 mice showed similar hCD45+ cell patterns with strong accumulation in lymphoid follicles at the periarteriolar lymphoid sheath and associated lymph follicles as well as in the parenchyma (**Figure 5**).

**Figure 6:**
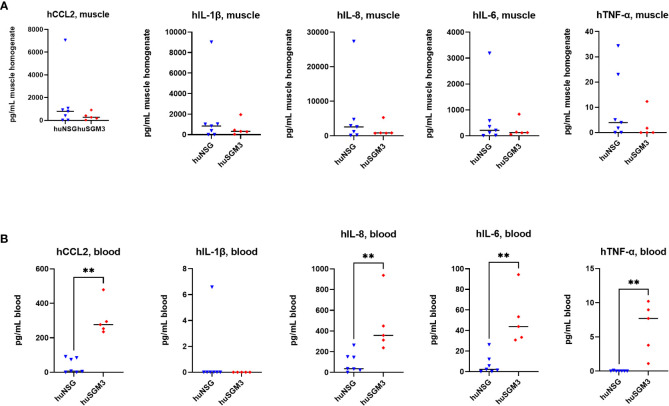
Levels of human cytokines in the infected thigh muscle **(A)** and the blood **(B)** of *S. aureus*-infected huNSG or huSGM3 mice on day 2 p.i. **(A)** The infected thigh muscles were recovered and homogenized in sterile PBS. Cytokine levels in filtered homogenate were then determined by a Luminex assay. **(B)** Blood serum was recovered on day 2 p.i. and the cytokine levels measured with a Luminex assay. Displayed are the individual values and the respective median per group. Statistical significance was tested with Mann-Whitney test (**p < 0.01).

Next, the levels of murine and human cytokines/chemokines CCL2, IL-1β, IL-6, IL-10 and TNFα, as well as of human IL-8 were determined in the infected thigh muscle and in the blood on d2 p.i., since we hypothesized that the higher immune cell numbers in huSGM3 might be accompanied by higher levels of effector molecules and an elevated inflammatory state. The levels of murine cytokines were similar for all groups in both compartments, indicating that the infection with *S. aureus* did not lead to different activation of the murine immune cells (**Figure S3**). The examination of human cytokine levels revealed that while the levels at the primary site of infection were similar for huSGM3 and huNSG mice, strongly increased levels of CCL2, IL-6, IL-8 and TNF-α could be measured by the Luminex assay in the blood (**Figure 6**). This indicates a systemic response of the human immune system in huSGM3 mice against the *S. aureus* infection and might furthermore reflect the response to systemically spreading bacteria.

### The stronger the humanization, the higher the vulnerability to *S. aureus* infection

**Figure 7:**
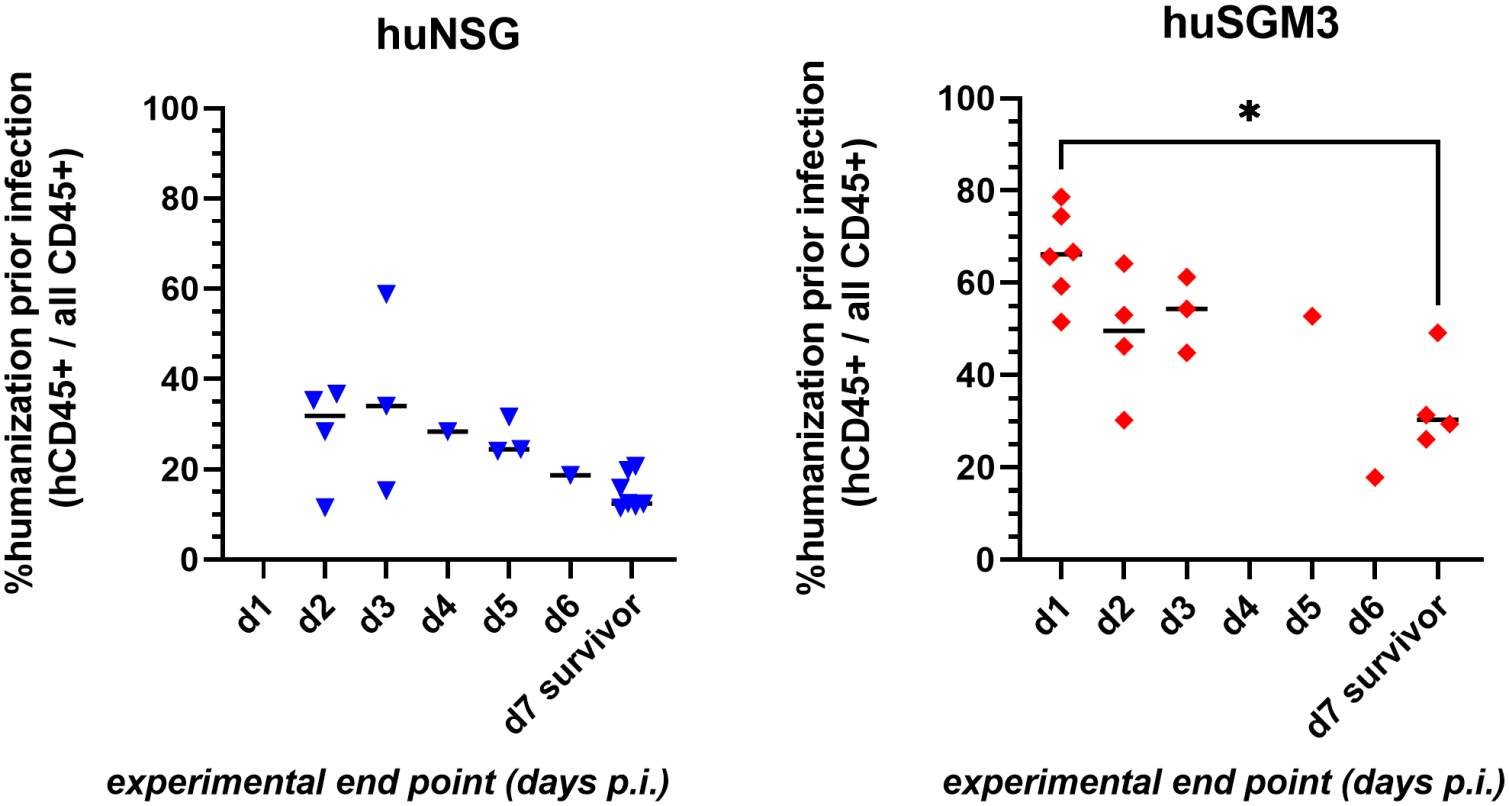
Correlation of rate of humanization prior infection and severity of disease during infection experiment. Blood was drawn from each individual mouse prior infection and analyzed by flow cytometry for the presence of human and murine CD45+ cells. The experimental end point was defined by either the mouse reaching the humane end point (according to the score sheet) or surviving until day 7 p.i. (when the experiment was ended). Statistically significant differences between points in time were calculated by Kruskal-Wallis with Dunn’s multiple comparison test (*: p < 0.05).

The decreased survival of huSGM3 mice following local *S. aureus* infection in the thigh muscle compared to huNSG mice was accompanied by higher human immune cell numbers in blood and spleen, as well as with increased levels of immune effector molecules in the blood, but not with increased bacterial burden. Thus, we hypothesized, that the vulnerability does not originate from the pathogen itself but rather from the human immune system. This leads in consequence to the assumption, that a higher rate of humanization might cause higher susceptibility against *S. aureus*. Comparing the rate of humanization (prior infection) to the time at which each individual mouse reached the humane end point (or survived until the end of the experiment on day 7 p.i.), showed a clear negative correlation between both parameters (**Figure 7**). In consequence, high numbers of human immune cells prove to be detrimental for the mice during bacterial infection with *S. aureus*.

## Discussion

Humanized mice, respectively mice with a humanized hemato-lymphoid system, emerged as a promising model to study infections and their treatment or prevention in recent years. Most commonly, they are generated by engrafting immunodeficient mice with human CD34+ stem cells, which repopulate hematopoietic niches and give rise to various human cell lineages within the mouse (10, 23). These models have proven their worth for the investigation of human-specific or human-adapted pathogens like HIV, *Salmonella enterica* subsp. *enterica* serovar Typhi or *Mycobacterium tuberculosis* (22, 35). *S. aureus* is not limited to its capability to infect humans, but can also infect other mammals or prosper as colonizer on different species (36, 37). However, it has become clear in the last decade, that those strains of *S. aureus* which are exceptionally successful in clinics, have evolved a large repertoire of virulence factors which can be regarded as highly human-specific (24, 38). The idea that these strains might behave differently when challenged with a human rather than with a murine immune system during infection, resulted in the application of humanized mice. This hypothesis was recently tested in different experimental models and it could be shown that humanized NSG mice are more susceptible to *S. aureus* infection than wild-type, murinized (immunodeficient mice engrafted with murine stem cells instead of human ones) and even non-reconstituted immunodeficient mice (12–16). These studies implemented disease models of peritonitis, pneumonia, osteomyelitis, skin and deep tissue infections, thus covering a wide range of the clinical manifestations of *S. aureus*. All but one (13) of the above-mentioned studies applied huNSG mice, which are widely used in the community but which have a poor reconstitution of the human myeloid immune cell compartment (17, 23). Since myeloid cells, particularly neutrophils, play a major role in the defense against *S. aureus* (24, 25, 39), we hypothesized that next-generation humanized mice with a stronger reconstitution of the myeloid compartment might even be better suited to investigate *S. aureus* infections. To test this hypothesis, we humanized NSG and NSG-SGM3 mice by administration of human cord blood derived CD34+ hematopoietic stem cells. The genetically integrated human SCF, GM-CSF and IL-3 genes enabled overall higher numbers of human CD45+ cells in the blood of NSG-SGM3 mice, particularly of hCD66b+ granulocytes, similarly to earlier publications (17, 40).

On the other hand, previous studies demonstrated that the expression of the human cytokines/growth factors in NSG-SGM3 mice at supraphysiological levels comes with side effects during humanization, namely a deficiency in hematopoiesis (41, 42) and the development of secondary hemophagocytic lymphohistiocytosis (HLH) and/or macrophage activation syndrome (MAS) (30–32). We, too, could observe hallmarks of a MAS/HLH-like disease starting at week 10 post engraftment, namely a decrease in erythrocyte numbers as well as of hemoglobin content and an increase of reticulocyte numbers in the blood of humanized NSG-SGM3 mice. In order to prevent that these physiological changes alter the outcome of a *S. aureus* infection experiment, we decided to infect huSGM3 mice at 12 weeks post hCD34+ administration with *S. aureus* when signs of anemia were still rather mild but the number of human immune cells significantly higher than in huNSG mice. In order to identify differences in the susceptibility to *S. aureus* infection, we included age-matched Balb/c, wild-type NSG-SGM3 and murinized NSG-SGM3 mice as well as humanized NSG mice at 18 weeks post stem cell administration.

When huSGM3 and huNSG as well as the control groups were infected with *S. aureus* into the left thigh muscle, we could observe two outcomes: (I) both humanized mouse groups developed systemic signs of disease and succumbed to the bacterial infection, while wild type, immunodeficient or murinized mice survived and could control the infection and (II) the next-generation huSGM3 mice were significantly more vulnerable than huNSG mice. Interestingly, the impaired survival of humanized mice was not accompanied by an increased bacterial burden in the infected thigh muscle or inner organs, suggesting that the detrimental outcome was not linked to bacteria overwhelming the immune system in the early phase of the infection. The comparison of the rate of humanization and the time at which each individual mouse reached the humane end point demonstrated a clear influence of the humanization on the outcome of the infection. This is in line with earlier studies demonstrating this correlation in huNSG mice (14, 16). On the other hand, the humanization of both NSG and NSG-SGM3 mice led to lower numbers of murine Ly6G+ neutrophils and Ly6C+ monocytes, which might as well have impacted the outcome of the infection. Since the number of murine cells was similar for huSGM3 and huNSG, we can assume that the difference in susceptibility between these two groups is not linked to the murine immune system. The repertoire and capability of the human immune system in huNSG and huSGM3 mice on the other hand appeared largely different. HuSGM3 mice had overall higher numbers of human CD45+ cells in the blood and the spleen with particularly higher numbers of myeloid cells. But interestingly, the increased number of human neutrophils in the blood of huSGM3 mice did neither help to control nor limit the bacterial infection compared to the huNSG mice, even though they play a decisive role in the human immune defense against *S. aureus* (24, 25, 39). The higher human immune cell content was accompanied by elevated levels of cytokine in the blood, suggesting strong activation of the human immune system in huSGM3 mice. Increased levels of IL-6, IL-8 and TNFα have been shown to be significantly increased in *S. aureus* patients compared to healthy controls (43) and are associated with a complicated course of infection in *S. aureus* bacteremia patients (44–46). Our data showed significantly higher levels of these cytokines in the blood of huSGM3 than in huNSG mice. This suggests, together with the decreased survival, that huSGM3 mice might reflect the clinical course of severe *S. aureus* infections closer than huNSG or wildtype mice and that they might be a promising model to study cytokine intervention therapy. On the other hand, further efforts to improve the current humanized mouse models might be necessary to close the gap between model and clinics. In particular, because both, huNSG and huSGM3, deviate from the clinical representation by some missing, underrepresented or immature immune cell lineages, impaired antigen-specific adaptive immunity or limited graft-to-host tolerance (23).

We can summarize, that both the increased human immune cell reconstitution, particularly of myeloid cells, and the stronger human immune response in huSGM3 mice failed to control or resolve *S. aureus* infection in this deep-tissue abscess model. Our study suggests on the contrary that the stronger humanization of huSGM3 mice had a detrimental effect on the survival after local infection with *S. aureus*. This might indicate the failure of the human immune system to fight *S. aureus* as efficiently as the murine one and/or the adaption of *S. aureus* to components of the human immune system. Furthermore, humanized mice might help to reveal the pathogenic potential of *S. aureus*, which is impaired during infection in wild-type mice since many of the deployed virulence factors have a high degree of human specificity. It might be very interesting for future studies to reveal whether *S. aureus* adapts its pathogenic strategy and gene expression when infecting humanized mice compared to wild type mice. Taken together, we could show that next-generation humanized mice are more vulnerable to *S. aureus* infection than previous mouse models and might help to understand in the future why and how *S. aureus* became one of the most successful pathogens in humans.

## Data availability statement

The original contributions presented in the study are included in the article/**Supplementary Material**. Further inquiries can be directed to the corresponding author.

## Ethics statement

The animal study was reviewed and approved by Government of Lower Franconia, Germany.

## Author contributions

TH, KO, SS, and JD contributed to conception and design of the study. SH, AK, FK, LD, EI and TH performed the experiments. SH, AK, FK, LD, EI and TH analyzed the data. TH and SH wrote the first draft of the manuscript. AK, FK, JD, EI, SS and KO wrote sections of the manuscript. All authors contributed to the article and approved the submitted version.
