# Supplementary material for: Next-generation humanized NSG-SGM3 mice are highly susceptible to Staphylococcus aureus infection
Source: Front Immunol. 2023 Mar 10;14:1127709. doi: 10.3389/fimmu.2023.1127709 (PMC10037040; doi:10.3389/fimmu.2023.1127709)
Supplement: Supplementary file 1 [file DataSheet_1.docx]

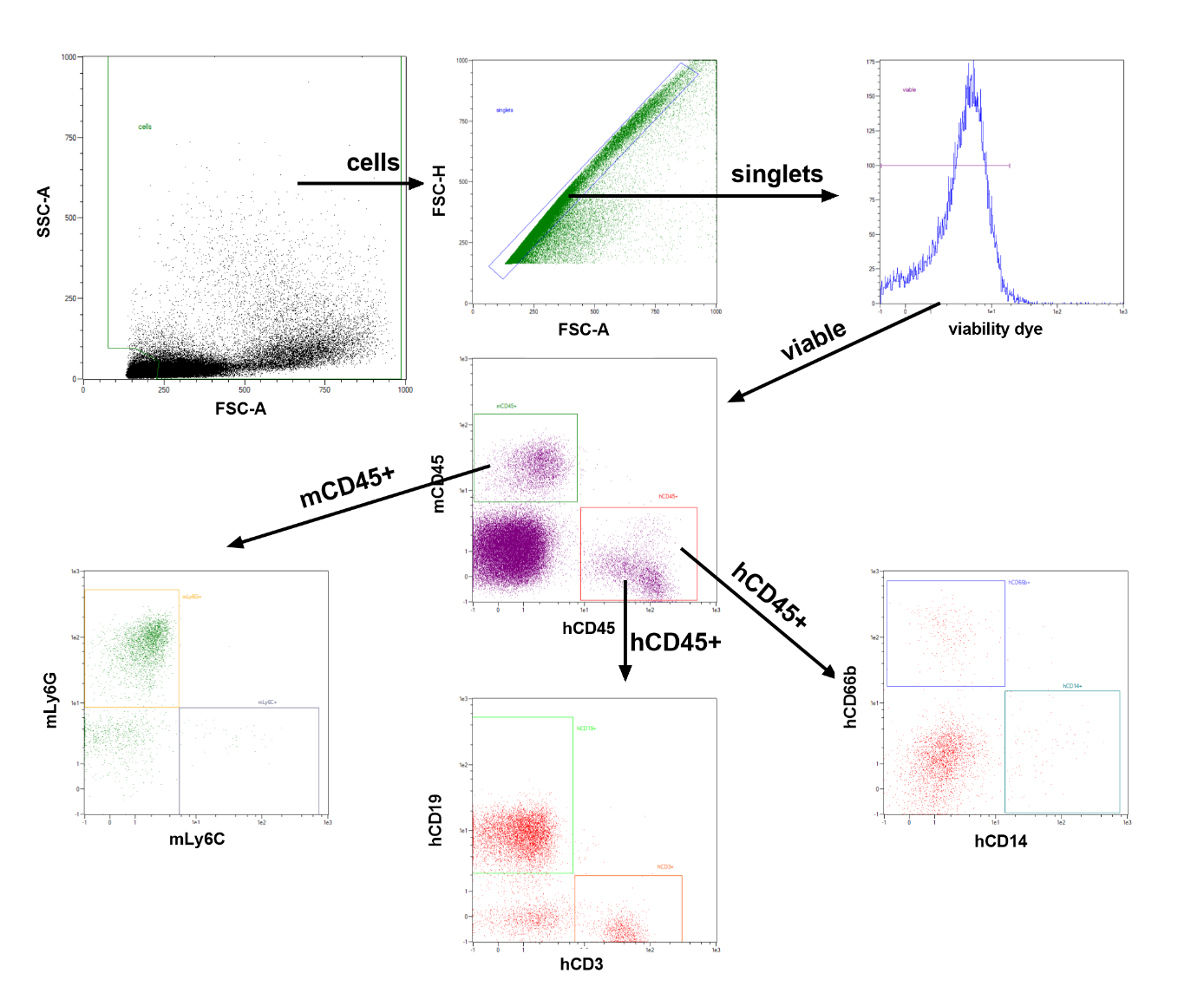


**Fig. S1: Schematic representation of the hierarchical gating strategy for the analysis of human and murine immune cells in blood, spleen and bone marrow.** Single cell suspensions were stained with viability dye and only viable singlets analyzed for their surface antigen composition.


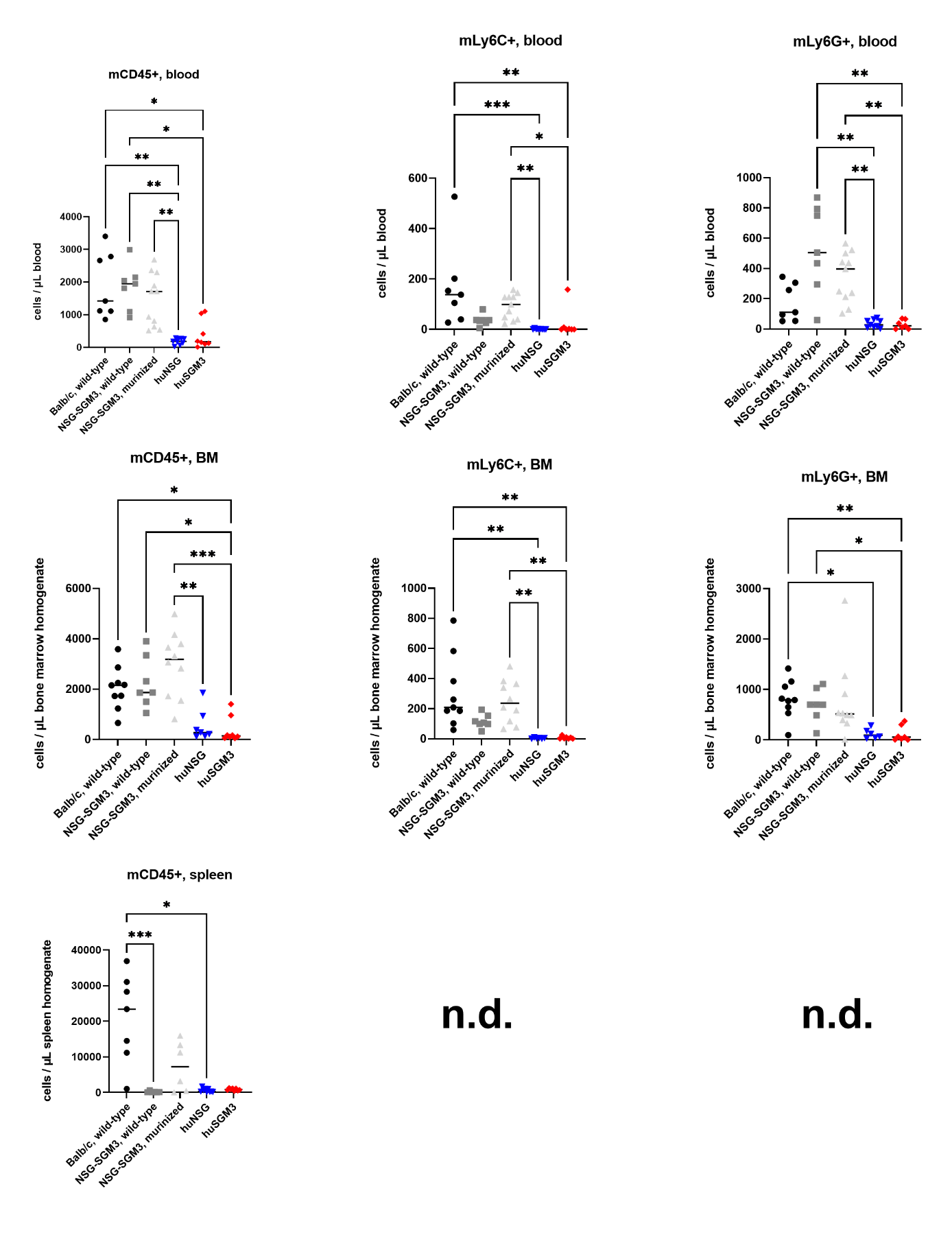


**Fig. S2:** **Murine immune cells in *S. aureus* infected huNSG and huSGM3 mice.** Immune cells were measured by flow cytometry with antibodies against hCD45, mCD45, mLy6C, and mLy6G. **(A)** Blood samples were analyzed at day 2 p.i.. **(B)** Bone marrow was harvested by flushing tibia and femur at day 2 p.i.. **(C)** Spleens were recovered at day 2 p.i. and homogenized by pressing through a 70 µm cell strainer. Displayed are the individual values per mouse as well as the medians per group. Statistical significance was tested with either Kruskal-Wallis with Dunn’s multiple comparison test (A) or Mann-Whitney-test (B + C) (*: p < 0.05, **: p < 0.01, ***: p < 0.005).


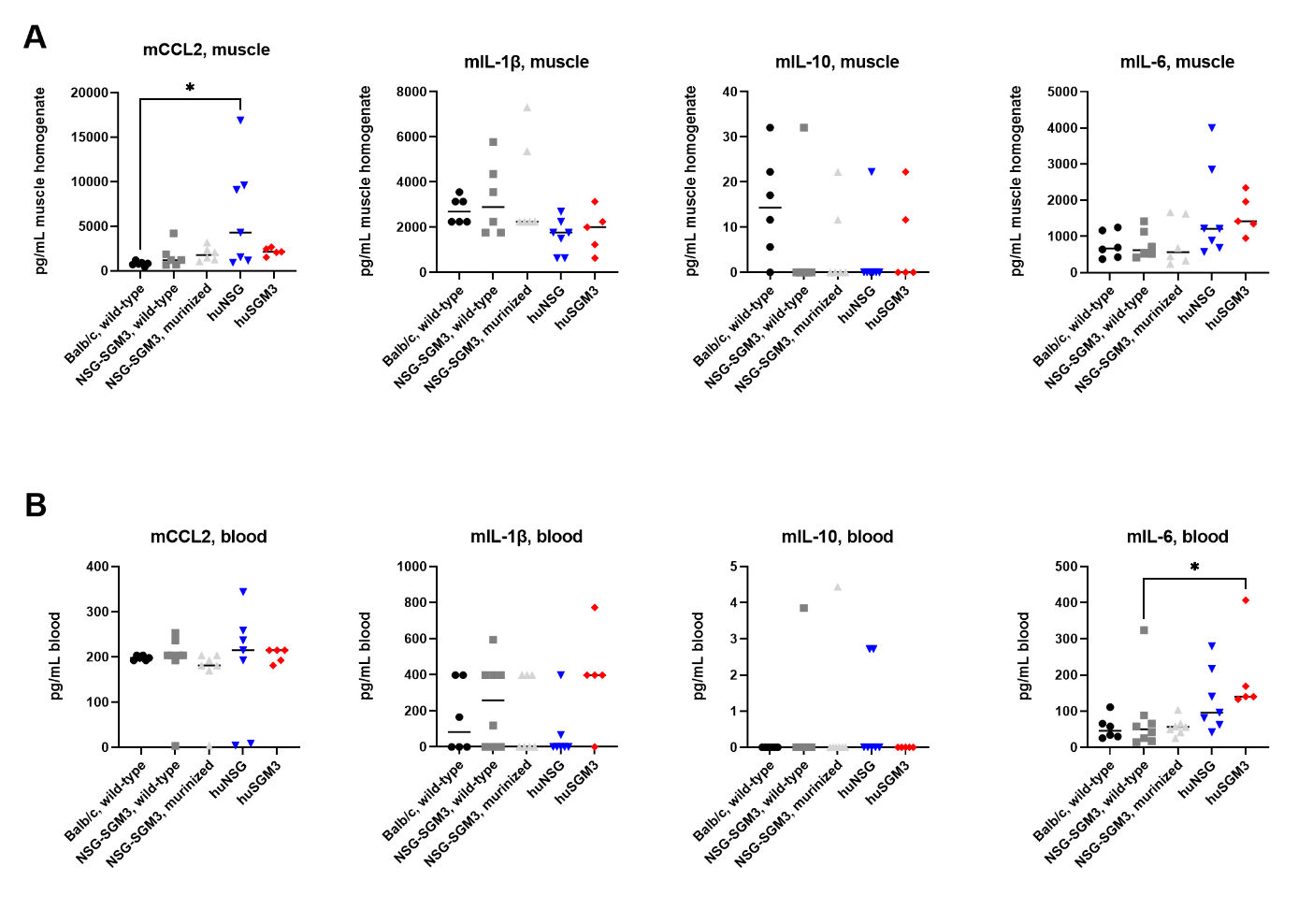


**Fig. S3:** **Levels of selected murine cytokines in the infected thigh muscle (A) and the blood (B) of *S. aureus* infected humanized, wild-type or unreconstituted mice at day 2 p.i..** **(A)** The infected thigh muscles were recovered and homogenized in sterile PBS. Cytokine levels in filtered homogenate were then determined by a Luminex assay. **(B)** Blood serum was recovered at day 2 p.i. and the cytokine levels measured with a Luminex assay. Displayed are the individual values and the respective median per group. Statistical significance was tested with Kruskal-Wallis with Dunn’s multiple comparison test (*: p < 0.05).
